# Cytoplasmic Ubiquitin-Specific Protease 19 (USP19) Modulates Aggregation of Polyglutamine-Expanded Ataxin-3 and Huntingtin through the HSP90 Chaperone

**DOI:** 10.1371/journal.pone.0147515

**Published:** 2016-01-25

**Authors:** Wen-Tian He, Xue-Ming Zheng, Yu-Hang Zhang, Yong-Guang Gao, Ai-Xin Song, Françoise Gisou van der Goot, Hong-Yu Hu

**Affiliations:** 1 State Key Laboratory of Molecular Biology, Institute of Biochemistry and Cell Biology, Shanghai Institutes for Biological Sciences, Chinese Academy of Sciences, Shanghai 200031, China; 2 Global Health Institute, Ecole Polytechnique Fédérale de Lausanne (EPFL), Lausanne, Switzerland; 3 Department of Biochemistry and Molecular Biology, School of Medical Technology, Jiangsu University, Zhenjiang 212013, Jiangsu, China; UMCG, NETHERLANDS

## Abstract

Ubiquitin-specific protease 19 (USP19) is one of the deubiquitinating enzymes (DUBs) involved in regulating the ubiquitination status of substrate proteins. There are two major isoforms of USP19 with distinct C-termini; the USP19_a isoform has a transmembrane domain for anchoring to the endoplasmic reticulum, while USP19_b contains an EEVD motif. Here, we report that the cytoplasmic isoform USP19_b up-regulates the protein levels of the polyglutamine (polyQ)-containing proteins, ataxin-3 (Atx3) and huntingtin (Htt), and thus promotes aggregation of their polyQ-expanded species in cell models. Our data demonstrate that USP19_b may orchestrate the stability, aggregation and degradation of the polyQ-expanded proteins through the heat shock protein 90 (HSP90) chaperone system. USP19_b directly interacts with HSP90 through its N-terminal CS (CHORD and SGT1)/P23 domains. In conjunction with HSP90, the cytoplasmic USP19 may play a key role in triage decision for the disease-related polyQ-expanded substrates, suggesting a function of USP19 in quality control of misfolded proteins by regulating their protein levels.

## Introduction

Protein homeostasis or proteostasis is vital to normal cellular growth and function, which can be perturbed by many factors, such as physiological stress or genetic mutation [1, 2]. Cells have evolved two kinds of elaborated systems, molecular chaperones and protein degradation machineries, for quality control of cellular proteins [3]. Molecular chaperones can recognize unfolded, misfolded or damaged proteins and facilitate their refolding to native conformations [4]. Irreversibly misfolded proteins may form aggregates and/or undergo degradation mainly through ubiquitin-proteasomal pathway [4]. In eukaryotic cells, whether a misfolded protein forms aggregates or has to be degraded is elaborately regulated by cross-talk between these two mechanisms, in which numerous adaptor proteins [5, 6], chaperones [7, 8] and/or co-chaperones [9] are closely associated. In particular, ubiquitination of substrates is counteracted by deubiquitination allowing escape of misfolded and aggregated proteins from degradation [10, 11]. On the other hand, the accumulation of insoluble aggregates may result in cell toxicity and ultimately the pathogenesis of several neurodegenerative diseases. Among these, polyglutamine (polyQ)-expanded ataxin-3 (Atx3) and huntingtin (Htt) are the main causative proteins of spinocerebellar ataxia type-3 (SCA3) and Huntington’s disease, respectively [12, 13].

Ubiquitin-specific protease 19 (USP19) is a member of the deubiquitinating enzyme (DUB) family, whose structure and function are largely unknown [14]. There are two major isoforms of USP19 that differ in their C-termini; one contains a transmembrane domain (TMD) for anchoring the protein to the endoplasmic reticulum (ER) and is involved in ER-associated degradation (ERAD) of substrates [15], the other has a C-terminal EEVD extension, putatively interacting with tetratricopeptide repeat (TPR)-containing proteins [16], such as carboxyl-terminus of Hsc70 interacting protein (CHIP) [17]. Interestingly, both forms of USP19 contain two CS (CHORD-SGT1)/P23 domains in their N-termini that potentially interact with the HSP90 chaperone [18], and a central USP domain that has the deubiquitinating activity [15]. Recently, both isoforms of USP19 have been verified in muscle cells by qPCR using isoform-specific primers [19]. Up to date, studies have focused on the ER-resident isoform of USP19 (USP19_a), which participates in the unfolded protein response and rescues the ERAD substrates [15, 20]. Besides, USP19 has also been proposed to play a role in regulating the stabilities of the ubiquitin ligase KPC1 [21], inhibitors of apoptosis c-IAP1 and c-IAP2 [22], and hypoxia inducible factor 1α (HIF-1α) during hypoxia [23].

As a multi-domain DUB, USP19 is implicated in regulating protein deubiquitination and triage decision, which might be closely associated with protein aggregation and degradation [24]. In this work, we studied the potential regulatory functions of USP19 on polyQ-containing proteins, Atx3 [9] and Htt [25]. We found that the cytoplasmic isoform USP19_b up-regulates the protein levels of these proteins and aggravates aggregation and cytotoxicity of the polyQ-expanded species depending on the HSP90 chaperone. Our findings support a role of USP19 in regulating the balance between aggregation and degradation of cellular polyQ-expanded proteins in the quality control [24].

## Materials and Methods

### Materials and expression plasmids

17-AAG (17-(Allylamino)-17- demethoxygeldanamycin) and MG132 were purchased from Sigma and Calbiochem, respectively. The antibodies against HA, FLAG and endogenous CHIP were obtained from Sigma, while those against HSP90 and Myc were from Cell Signaling and those against GFP, ubiquitin and actin from Santa Cruz. The anti-USP19 antibody (A301-587A) was purchased from Bethyl Laboratories. The goat anti-mouse IgG-HRP antibody, goat anti-rabbit IgG-HRP, rabbit anti-goat IgG-HRP secondary antibodies and FITC-conjugated anti-mouse antibody, and Cyanine 3 conjugated anti-rabbit secondary antibody were purchased from Jackson Immuno-Research. The proteins were visualized using an ECL detection kit (Amersham Pharmacia Biotech). CytoTox-ONE^™^ reagent was a product of Promega. Human USP19_a, USP19_b, USP5 and all the mutants were cloned into HA-pcDNA3 vector. The mutants (C506S, CS1M, CS2M, CS12M, ΔN393) of USP19_b, C335A mutant of USP5 and the Atx3_100Q_-UIM^mut^ (S236A/S256A) mutant were generated using site-directed mutagenesis via PCR technique. The expression plasmid for human HSP90 was cloned into pcDNA3.1-Myc/His, and the pEGFP-N1 vector was used to express EGFP. The polyQ proteins (including Atx3_22Q_, Atx3_100Q_, Atx3_100Q_-UIM^mut^, Htt-N552_18Q_, Htt-N552_100Q_ and Htt-N171_100Q_) and TDP-35 were cloned into pcDNA3-FLAG plasmid.

### Cell culture and transfection

Human HEK 293T cells were cultured in Dulbecco’s modified Eagle’s medium (DMEM) (Invitrogen) supplemented with 10% fetal bovine serum (Gibco) and grown at 37°C under a humidified atmosphere containing 5% CO_2_. All the plasmid transfections were performed using Fugen (Roche) or PolyJetTM (SignaGen Laboratories) reagent following the manufacturer’s instructions.

### Immunofluorescence imaging

Cells were seeded onto poly-L-lysine hydrobromide-coated coverslips and transfected under a low confluence. After culturing for another 36–48 hrs, cells were harvested for immunofluorescence assay. Briefly, the cells were washed with a PBS buffer (10 mM Na_2_HPO_4_, 1.8 mM KH_2_PO_4_, 140 mM NaCl, 2.7 mM KCl, pH 7.3) three times and fixed with 4% paraformaldehyde for 30 min, rinsed and permeabilized with the PBS plus 0.1% Triton X-100, blocked in 3% bovine albumin, and then stained with primary antibody for 1 hr at room temperature. After washing the unbound antibody, FITC or TRITC conjugated secondary antibody was used to label the protein. The nuclei were stained with Hoechst (Sigma) and the ER was imaged with an antibody against calnexin. Fluorescence imaging was carried out by using a Leica TCS SP2 confocal microscope (Leica Microsystems).

### Co-immunoprecipitation and Western blotting

After transfection and cultivation for 24–48 hrs, HEK 293T cells were lysed in RIPA buffer (50 mM Tris–HCl, pH 7.5, 150 mM NaCl, 1 mM EDTA, 1 mM PMSF, cocktail protease inhibitor (Roche), 1% NP-40, 0.05% SDS) and the whole cell lysates were subjected to SDS-PAGE with 12% acrylamide gel and then transferred onto PVDF membranes (PerkinElmer). For co-IP experiments, the cell lysates were centrifuged at 13,000 g for 10 min, and the supernatants were mixed with protein A/G beads conjugated with appropriate specific primary antibodies. After incubating for 2–4 hrs at 4°C, the beads were washed with the lysis buffer for three times to remove the unbound proteins, and the precipitated proteins were subjected to immunoblotting analysis. The blots were incubated with appropriate specific antibodies and horseradish peroxidase-conjugated anti-IgG secondary antibody. The proteins were visualized using an ECL detection kit (Amersham Pharmacia Biotech). The band intensity was quantified by using *Scion Image*, and its integral area of gray value was calculated and normalized to that of the control. Data were statistically analyzed with one-way ANOVA.

### Supernatant/pellet fractionation

The cell lines over-expressing Atx3 transfected with USP19_b or its mutants were lysed with RIPA buffer on ice for 30 min and centrifuged at 16,000 g for 10 min. The pellet was sufficiently washed with RIPA buffer for three times and the cytosolic supernatant were added with equal amount of 2 x loading buffer (4% SDS) and subjected to Western blotting.

### Filter trap experiment

The filter trap experiment was performed as described previously [26] for examining protein aggregation in cell lysates. Cells were harvested and lysed in RIPA buffer (50 mM Tris–HCl, pH 7.5, 150 mM NaCl, 1 mM EDTA, 1% NP-40, protease inhibitor cocktail (Roche)). An equal volume of SDS buffer (4% SDS, 100 mM DTT) was added to the total lysates and boiled at 100°C for 5 min. The mixture was centrifuged at 12,000 rpm for 5 min and the supernatant was filtered through a cellulose acetate membrane (0.2 μm pore size, Whatman). The membrane was washed 2 times with 2% SDS and the aggregates retained on the membrane were detected with anti-FLAG antibody.

### GST pull-down experiment

The His-tagged HSP90 (in pET-28a) and GST-fused CS1 (residues 75–209) or CS2 (273–393) domain of USP19_b (in pGEX-4T-3) were expressed in *E*. *coli* BL21 (DE3) strain. The His-tagged HSP90 was purified through a Ni^2+^-NTA column (Qiagen), while GST-fused proteins were purified using the glutathione Sepharose 4B column (Amersham Bioscience). GST or GST-fused proteins were incubated with the glutathione Sepharose 4B beads in a PBS buffer (10 mM Na_2_HPO_4_, 140 mM NaCl, 2.7 mM KCl, 1.8 mM KH_2_PO4, pH 7.4), and the suspension was agitated at 4°C for 30 min. The beads were washed three times in the same buffer to remove any unbound protein. An equal molar amount of HSP90 was added, and the suspension was agitated at 4°C for about 2 hrs. After excessive washing, the beads were re-suspended in the sample buffer and subjected to SDS-PAGE followed by Coomassie staining.

### RNA interference

For knockdown of human endogenous USP19, three different siRNA duplexes (#1, SI00758163; #2, SI00758170; #3, SI00758177) were purchased from Qiagen. The target sequence of the viral glycoprotein VSVG (*ATTGAACAAACGAAAGGA*) was used for a control. Transfection of the siRNAs was performed using Lipofectamine^™^ (Invitrogen) or INTERFERin (Polyplus-transfection) transfection reagent with a dish of 100 pmol/6 cm^2^ of siRNA according to the manufacturer’s instruction. The cells were harvested for assays after 72 hrs.

### Cell viability assay [27]

HEK 293T cells transfected with equal amount of Atx3100Q or Htt-N171_100Q_ and USP19_b or its C506S mutant were plated on a 96-well dish and cultured for another 48 hrs in 100-μL medium. The cytotoxicities of polyQ proteins were measured using the CytoTox-ONETM (Promega) assay based on LDH release according to the manufacturer’s instruction. The fluorescence was recorded on Berthold LB940 with the excitation wavelength of 540 nm and an emission wavelength of 590 nm. The relative cytotoxicity was estimated by subtracting the background value, and the data were statistically analyzed with one-way ANOVA and presented as Mean ± SD.

## Results

**Fig 1:**
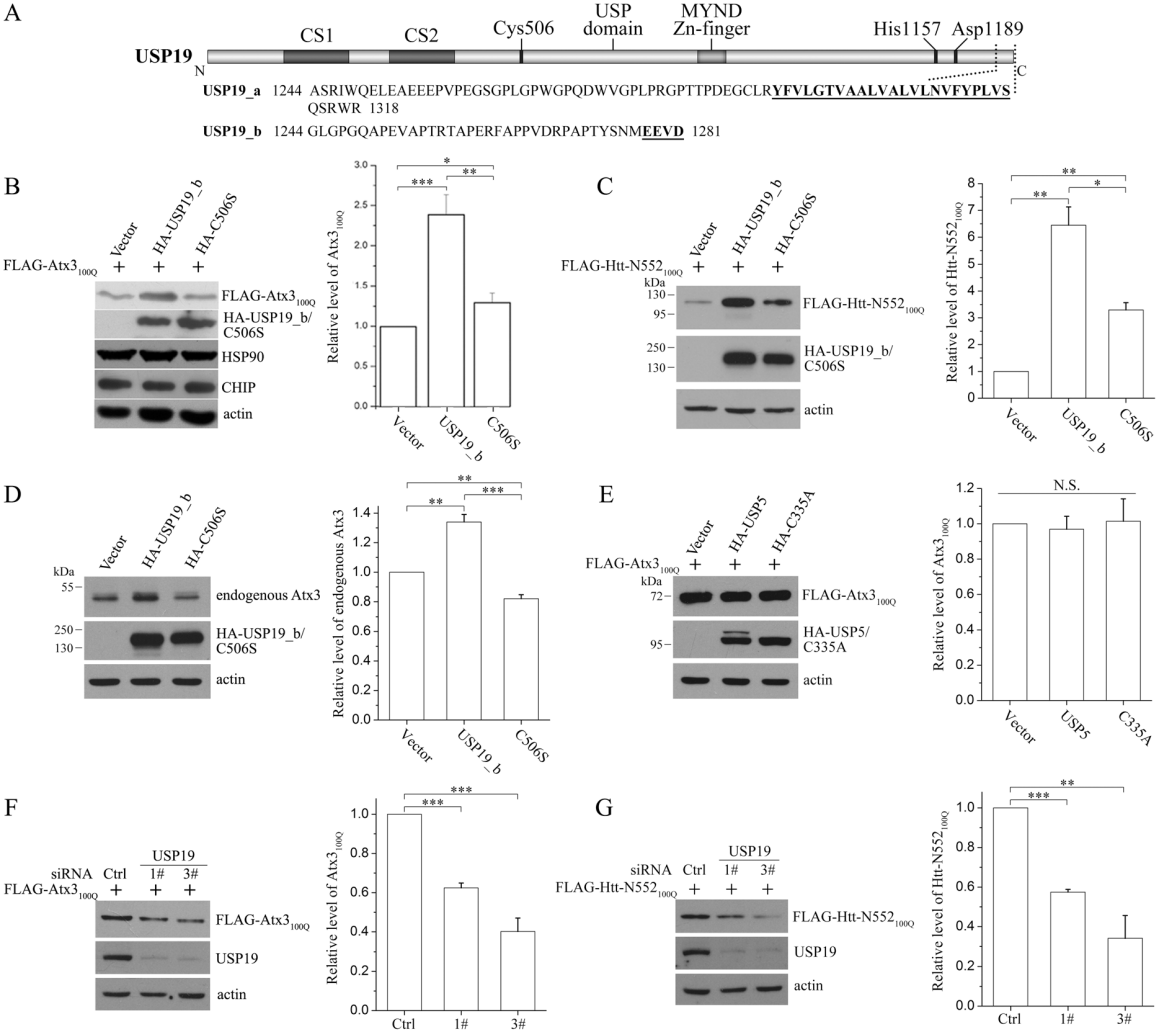
USP19_b increases the protein levels of Atx3_100Q_ and Htt-N552_100Q_. **A**, Domain architecture and sequence alignment of USP19 isoforms. USP19 contains two CHORD-SGT1 domains (namely CS1 and CS2) at its N-terminus and a large USP domain. Instead of a transmembrane domain in USP19_a, there is a relatively hydrophilic region and an EEVD motif in the C-terminus of USP19_b. **B** and **C**, Effects of USP19_b on the protein levels of Atx3_100Q_ (**B**) and Htt-N552_100Q_ (**C**). HEK 293T cells were transfected with equal amount of FLAG-tagged Atx3_100Q_ or Htt-N552_100Q_ and vector, HA-USP19_b or its C506S mutant, and 48 hrs later, the cells were harvested and lysed for Western blotting with the indicated antibodies. **D,** Effect of USP19_b on the protein level of endogenous Atx3. **E**, Effect of USP5 on the protein level of Atx3_100Q_. FLAG-tagged Atx3_100Q_ was co-transfected with HA-USP5 or its active-site mutant (C335A) into HEK 293T cells. **F** and **G**, Knockdown of USP19 reduces the protein level of Atx3_100Q_ (**F**) or Htt-N552_100Q_ (**G**) in HEK 293T cells. Cells were transfected with FLAG-tagged Atx3_100Q_ or Htt-N552_100Q_ and USP19 siRNA. After 72 hrs, the cells were harvested and the lysates were subjected to Western blotting with the indicated antibodies. Ctrl, VSVG siRNA; 1#, 3#, two siRNAs against USP19. The band intensities were quantitated by using *Scion* Image. Data were normalized to mock transfected with vector or control siRNA, and statistically analyzed with one-way ANOVA and presented as Mean ± SEM (n = 3). *, p < 0.05; **, p < 0.01; ***, p < 0.001; N.S., no significance.

Human *usp19* gene is located in chromosome 3 (3p21.31) and can be transcribed and translated into two major isoforms [28]. Isoform USP19_a contains a C-terminal TMD region and is anchored to endoplasmic reticulum [15]. Sequence alignment illustrates that the C-terminal region of USP19_b is characterized by an EEVD motif but without hydrophobic TMD (Fig 1A). Our immunofluorescence microscopic experiment confirmed the previous observation that USP19_a is an ER-anchored protein co-localizing with calnexin (an ER marker) (S1A Fig). In contrast, USP19_b did not co-localize with calnexin while showing a cytoplasmic staining (S1B Fig), as observed for USP19_a upon deletion of the C-terminal TMD [15].

### USP19_b up-regulates the protein levels of Atx3 and Htt-N552

We applied isoform I of Atx3 and N-terminal Htt (Htt-N552) as polyQ-containing substrates to study the potential regulatory functions of USP19 (S2 Fig). We co-transfected USP19_b or its active-site mutant (C506S) with polyQ-expanded Atx3 (Atx3_100Q_) into HEK 293T cells and analyzed the amounts of Atx3_100Q_ in cell lysates by Western blotting. The result showed that USP19_b significantly increased the protein level of Atx3_100Q_ in a manner dependent on the ubiquitin-specific protease activity (Fig 1B). Moreover, the increase of Atx3_100Q_ led by USP19_b was dose-dependent (S3A Fig). However, expression of USP19_b did not affect the levels of HSP90 and CHIP, two chaperones involved in quality control of misfolded proteins (Fig 1B, lower rows). Similarly, USP19_a could also up-regulate the protein level of Atx3_100Q_ in a dose-dependent manner (S3A Fig, right panels), suggesting that the C-terminal EEVD sequence of the b-form and the ER anchor location of the a-form are not essential to this effect. Similar results were also observed when co-transfection of USP19_b with Htt-N552_100Q_ that USP19_b up-regulated the protein level of Htt-N552_100Q_, whereas the C506S mutant had attenuated the effect (Fig 1C). It is noteworthy that the C506S mutant can partially increase the protein level of Atx3_100Q_ and Htt-N552_100Q_, but this effect is relatively small as compared with that of the wild type. We speculate that the C506S mutation does not abolish the activity of USP19_b completely, which is consistent with the previous observation that the C506S mutant of USP19_a has partial activity [15]. We also examined whether USP19_b exerts regulatory functions on normal polyQ-length proteins. Similar regulatory effect by USP19_b was observed in overexpressed Atx3_22Q_ (S3B Fig) and Htt-N552_18Q_ (S3C Fig). Furthermore, USP19_b could also up-regulate the protein level of endogenous Atx3 (Fig 1D), albeit this increasing effect was not obvious as that on the overexpressed Atx3_22Q_ (S3B Fig).

To exclude the possibility that other DUB could regulate the levels of these two polyQ proteins, we examined USP5, a well-studied DUB with relatively strong deubiquitination activity [10, 29]. The data showed that neither USP5 nor its C335A mutant presented such effects on the protein levels of Atx3_100Q_ (Fig 1E) and Htt-N552_100Q_ (S4A Fig). Moreover, we applied GFP as a control to eliminate the possible artifacts caused by overexpression, and found that USP19_b had no effect on the GFP level (S4B Fig). We also examined the possible role of USP19_b on TDP-35, a C-terminal 35-kDa fragment of TDP-43, which forms cytoplasmic inclusions or aggregates and is implicated in the pathogenesis of amyotrophic lateral sclerosis [30]. The data showed that USP19_b did not increase the TDP-35 level (S4C Fig).

We next examined the effect of *usp19* silencing on the protein levels of Atx3_100Q_ and Htt-N552_100Q_, and observed that knockdown of USP19 significantly reduced the protein levels of Atx3_100Q_ (Fig 1F) and Htt-N552_100Q_ (Fig 1G) both in HEK 293T cells and in human retinal pigment epithelial (RPE1) cells (S3D Fig). Collectively, these data illustrate that USP19_b up-regulates the protein levels of the polyQ-containing proteins.

### USP19_b promotes aggregation of polyQ-expanded Atx3 and Htt-N552

**Fig 2:**
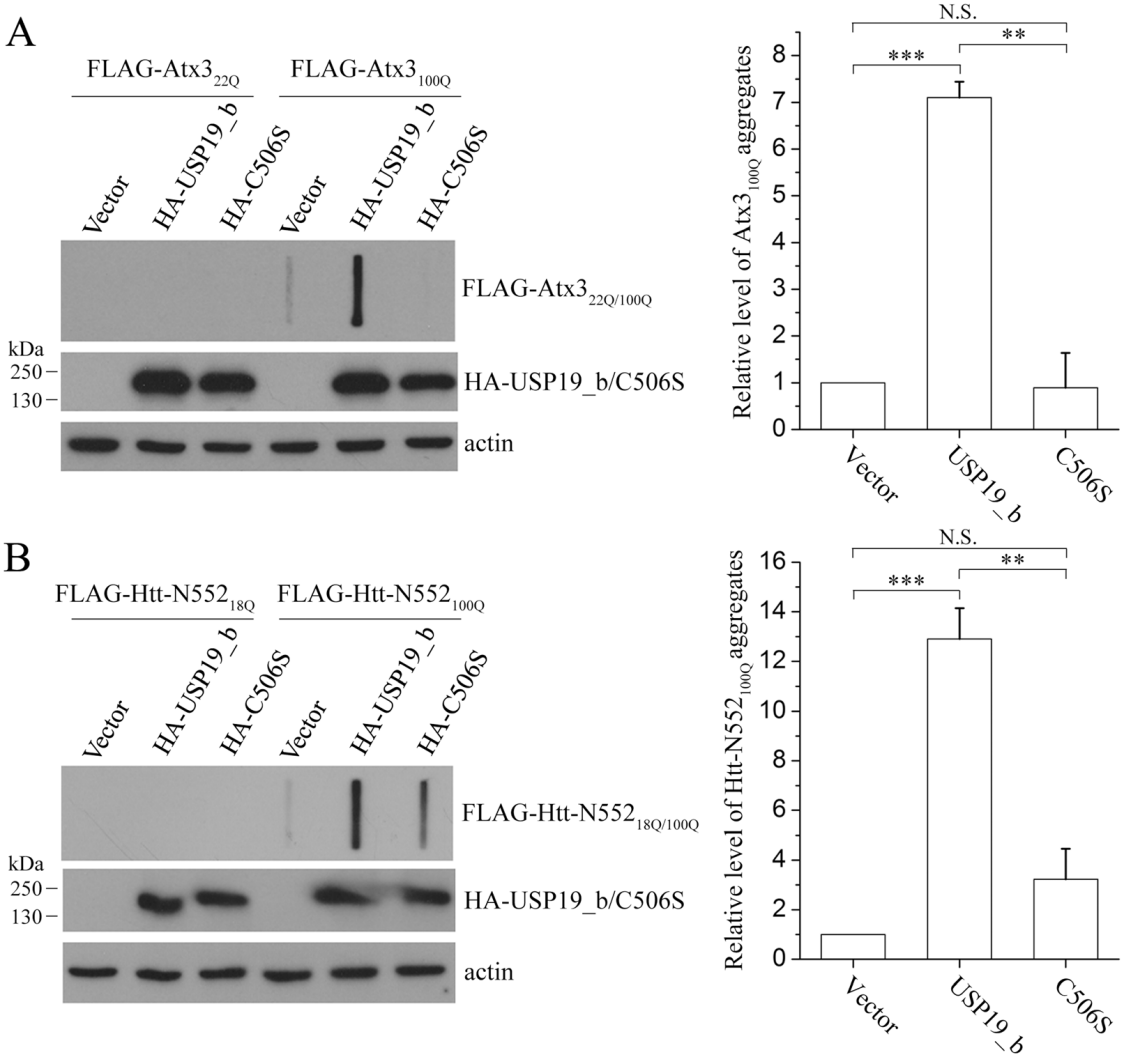
USP19_b promotes aggregation of Atx3_100Q_ and Htt-N552_100Q_ as evidenced by filter trap analysis. FLAG-tagged Atx3_100Q_ (**A**) or Htt-N552_100Q_ (**B**) was co-transfected with USP19_b or its C506S mutants into HEK 293T cells. Atx3_22Q_ and Htt-N552_18Q_ were set as controls. About 48 hrs after transfection, the cell lysates were subjected to filter trap and Western blotting analyses with an anti-FLAG antibody. Data were quantitated with the relative band intensities and presented as Mean ± SEM (n = 3). **, p < 0.01; ***, p < 0.001; N.S., no significance.

To get insights into whether USP19_b affects the soluble or aggregated form of polyQ-expanded proteins, we firstly analyzed the supernatant and pellet fractions of cell lysates and found that overexpression of USP19_b increased the amounts of the pellet fraction of Atx3_100Q_ as well as the supernatant (S5 Fig). It suggests that USP19_b up-regulates the Atx3_100Q_ level, while increase of the protein amount leads to aggregation. We then detected the aggregates by using filter trap experiments [26, 31]. The data showed that USP19_b could increase the amounts of SDS-resistant aggregates both in Atx3_100Q_ (Fig 2A) and Htt-N552_100Q_ (Fig 2B), but the C506S mutant had little effect on aggregate formation, although the proteins with normal polyQ lengths (Atx3_22Q_, Htt-N552_18Q_) did not form SDS-resistant aggregates (Fig 2, left panels). This implies that active USP19_b stimulates aggregation of the polyQ-expanded proteins in cells through up-regulating their protein levels.

**Fig 3:**
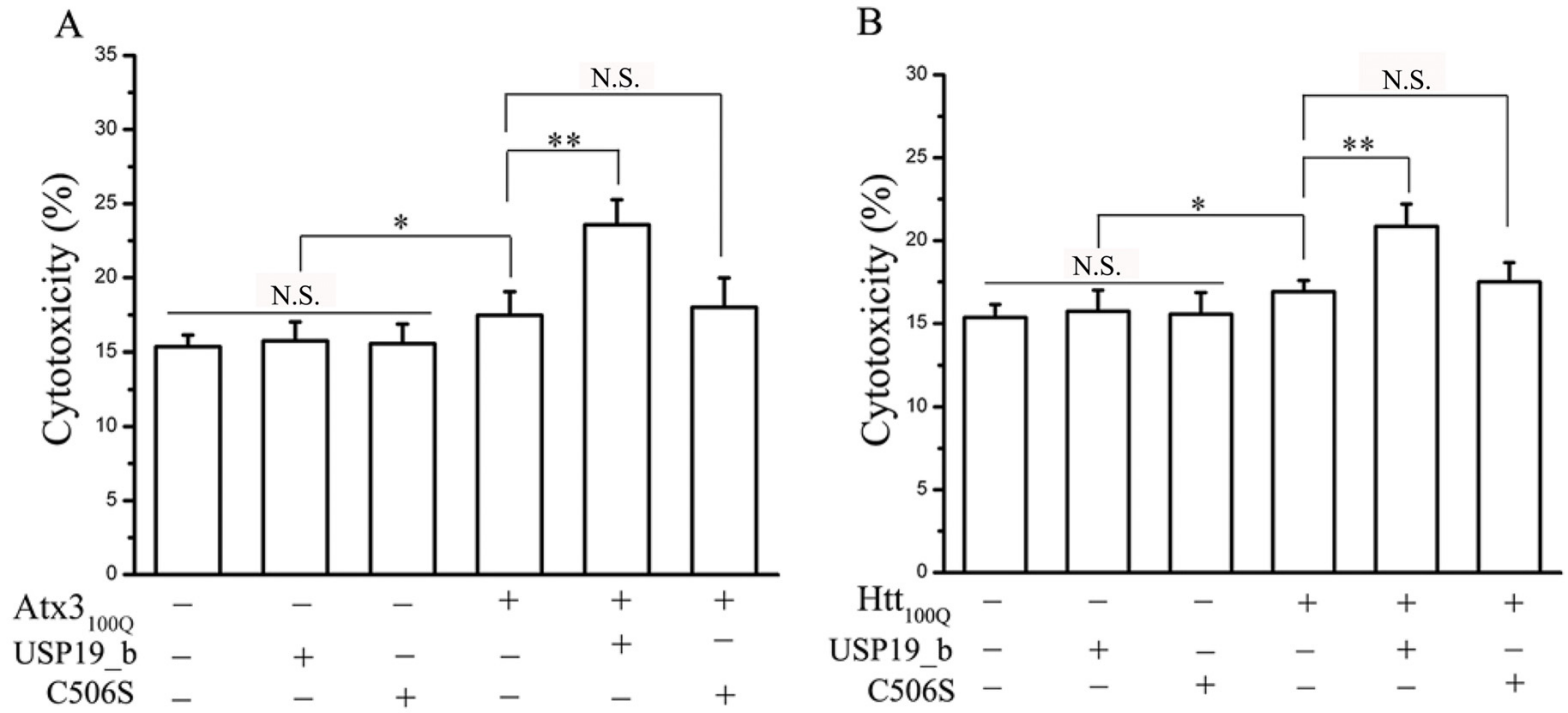
USP19_b stimulates the cytotoxicities of polyQ-expanded Atx3_100Q_ (A) and Htt-N171_100Q_ (B). Here Htt-N171_100Q_ refers to the N-terminal 171-residue fragment of Htt with 100Q. HEK 293T cells were co-transfected with the plasmids as indicated. After 48-hour culture, the cells were subjected to CytoTox-ONE^™^ assay. Data were statistically analyzed with one-way ANOVA and presented as Mean ± SD (n = 6). *, p < 0.05; **, p < 0.01; N.S., no significance.

As demonstrated, polyQ-expanded proteins, a culprit of related neurodegeneration, are susceptible to adopt non-native misfolded conformations that eventually form toxic oligomers and aggregates [32, 33]. We therefore analyzed the cytotoxicities of Atx3_100Q_ and Htt-N171_100Q_ in the context of overexpressed USP19_b in HEK 293T cells. As expected, neither USP19_b nor its C506S mutant alone affected the cytotoxicity, whereas overexpression of Atx3_100Q_ or Htt-N171_100Q_ led to an increase in toxicity (Fig 3). The cytotoxicities caused by the polyQ-expanded proteins were significantly enhanced by co-expression of wild-type USP19_b but not the C506S mutant. It indicates that the cytotoxicities of the polyQ-expanded proteins are associated with the deubiquitinating activity of USP19_b. Thus, USP19_b aggravates the cytotoxicities of the polyQ-expanded proteins by increasing their protein levels and possibly aggregate fractions.

### USP19_b interacts with HSP90 via its CS domains

**Fig 4:**
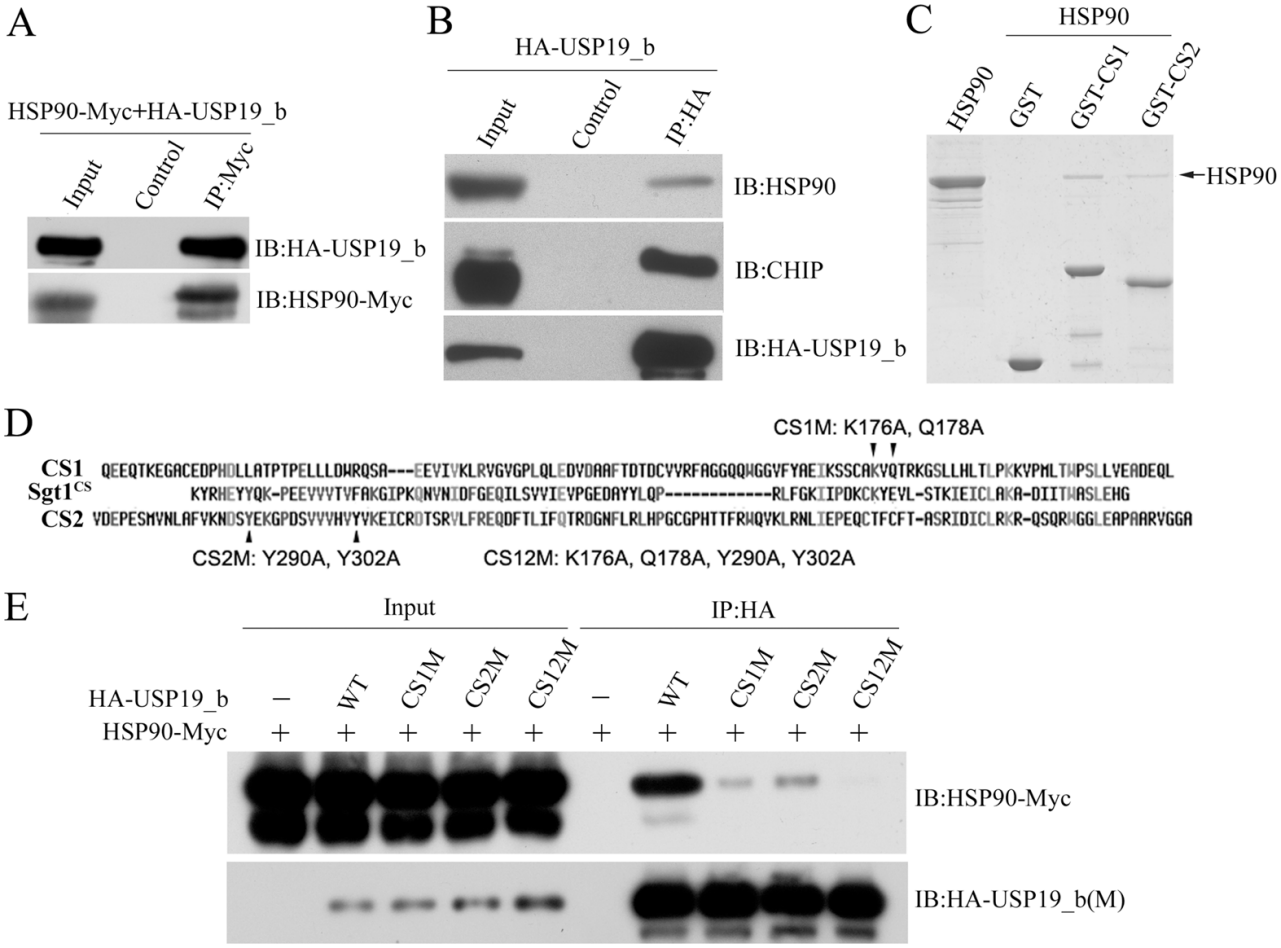
USP19_b associates with HSP90 through CS domains. **A**, Co-IP experiment showing interaction of USP19_b with HSP90. HEK 293T cells were co-transfected with HA-USP19_b and HSP90-Myc plasmids, and then the cell lysates were subjected to immunoprecipitation with protein A/G-conjugated anti-Myc antibody. The control lane is in the presence of only protein A/G. **B**, Immunoprecipitation with the endogenous HSP90 and CHIP by USP19_b. HEK 293T cells were transiently transfected with HA-USP19_b expression plasmid. **C**, GST pull-down experiment showing direct interaction between CS1 (residues 75–209) or CS2 (273–393) domain of USP19_b and HSP90. GST protein was set as a control. The arrow indicates the band of HSP90. **D**, Sequence alignment of the CS domains from USP19 (*Homo Sapiens*) and Sgt1a (*Arabidopsis*). The conserved residues that are putatively important to HSP90 binding were selected for mutation. **E**, Co-IP experiment of USP19_b or its CS-domain mutants with HSP90. CS1M, K176A/Q178A in the CS1 domain; CS2M, Y290A/Y302A in the CS2 domain; CS12M, double-domain mutant. HSP90 was Myc tagged, while USP19_b and its mutants were HA tagged.

Because USP19_b contains two CS domains (Fig 1A) potentially interacting with HSP90 [18], we attempted to elucidate whether USP19 regulates misfolded proteins through the HSP90 chaperone system [7]. We firstly investigated whether USP19_b interacts with HSP90. In the co-transfected HEK 293T cells, USP19_b could be immunoprecipitated by HSP90 (Fig 4A). A similar experiment also showed that overexpressed USP19_b could immunoprecipitate the endogenous HSP90 as well as CHIP proteins (Fig 4B). However, inconsistent with the recent data from immunoprecipitation experiment [20], GST pull-down showed that both CS1 and CS2 domains of USP19_b directly interacted with HSP90 (Fig 4C).

Based on sequence comparison and the complex structure of Sgt1a CS domain with HSP90 [18] (Fig 4D), we mutated two conserved binding-site residues on the CS1 (CS1M, K176A/Q178A) and CS2 (CS2M, Y290A/Y302A) domains, respectively. Co-IP experiment demonstrated that the CS mutants, especially the double-domain mutant (CS12M), significantly attenuated the interaction between USP19_b and HSP90 to different extents (Fig 4E). These data strongly indicate that USP19_b directly interacts with HSP90 through its CS domains. Collectively, the cytoplasmic USP19 associates with HSP90 potentially forming a dynamic complex in cells, which may function in quality control for the polyQ-expanded proteins.

### USP19_b modulates aggregation of the polyQ-expanded proteins through the HSP90 chaperone system

**Fig 5:**
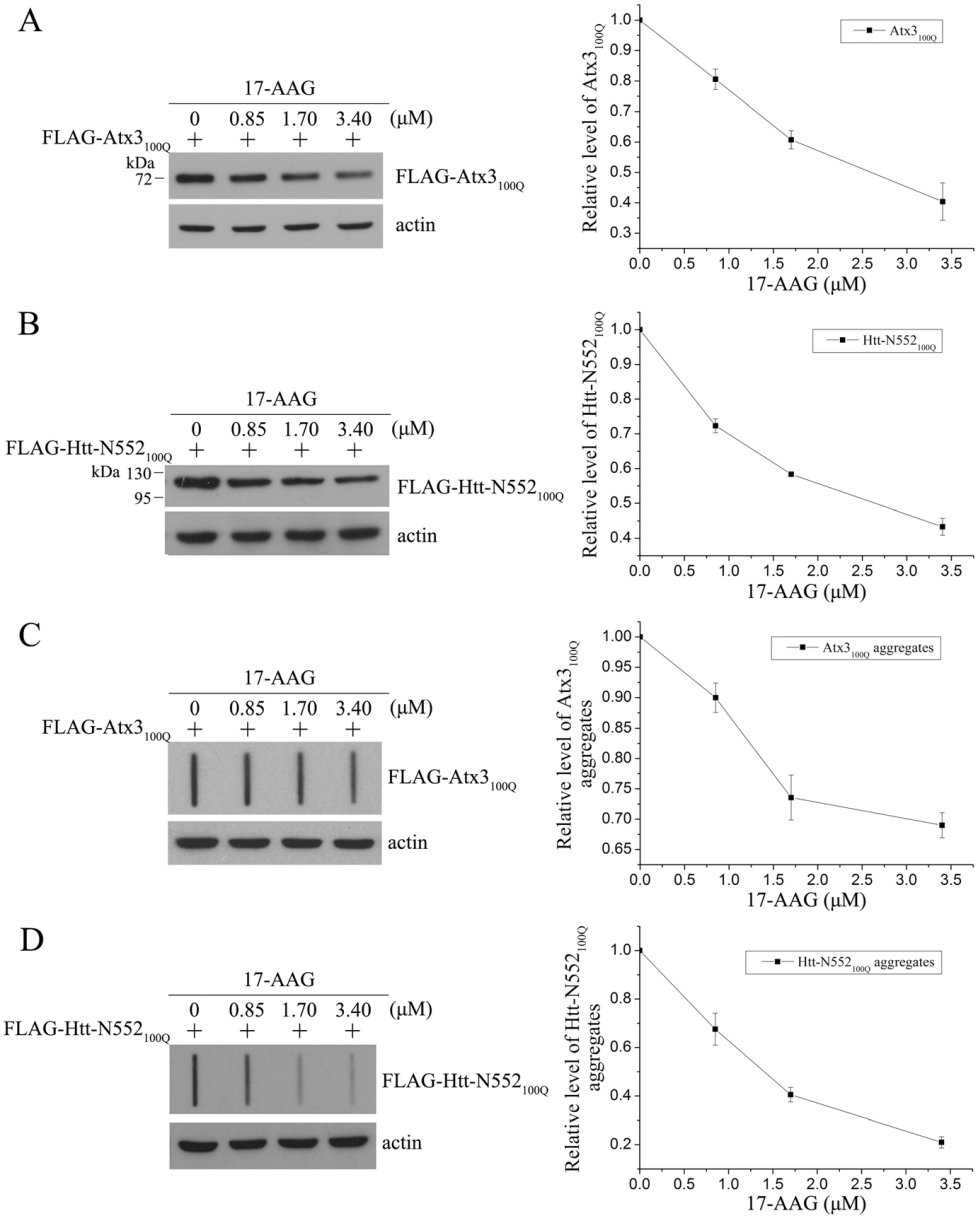
Inhibition of HSP90 down-regulates the protein levels and aggregates of Atx3_100Q_ and Htt-N552_100Q_. **A** and **B**, Effects of HSP90 inhibitor on the protein levels of Atx3_100Q_ (**A**) and Htt-N552_100Q_ (**B**). FLAG-tagged Atx3_100Q_ or Htt-N552_100Q_ was transfected into HEK 293T cells, and then the cells were treated with different doses of 17-AAG for 6 hrs (DMSO as a control). About 48 hrs after transfection, the cells were harvested and lysed for Western blotting. **C** and **D**, Effects of HSP90 inhibitor on the SDS-resistant aggregates of Atx3_100Q_ (**C**) and Htt-N552_100Q_ (**D**) by filter trap analysis. Data were quantitated with relative band intensities and presented as Mean ± SEM (n = 3).

It was reported previously that, as normal Atx3 [34], the polyQ-expanded proteins are mainly degraded through the ubiquitin-proteasome pathway [9, 34]. To ask whether HSP90 is involved in the USP19_b functionality for regulating polyQ-expanded substrates, we applied an HSP90 inhibitor 17-AAG and detected its effects on the substrates [35]. When the cells overexpressing a polyQ-expanded protein were treated with 17-AAG, the total amounts of Atx3_100Q_ (Fig 5A) and Htt-N552_100Q_ (Fig 5B) were decreased considerably. We also detected the SDS-resistant aggregates by filter trap assay; the aggregates of both Atx3_100Q_ (Fig 5C) and Htt-N552_100Q_ (Fig 5D) were also decreased with the increase of 17-AAG. It suggests that HSP90 is involved in stabilizing the polyQ-expanded proteins or functions in accumulation of the misfolded aggregates.

**Fig 6:**
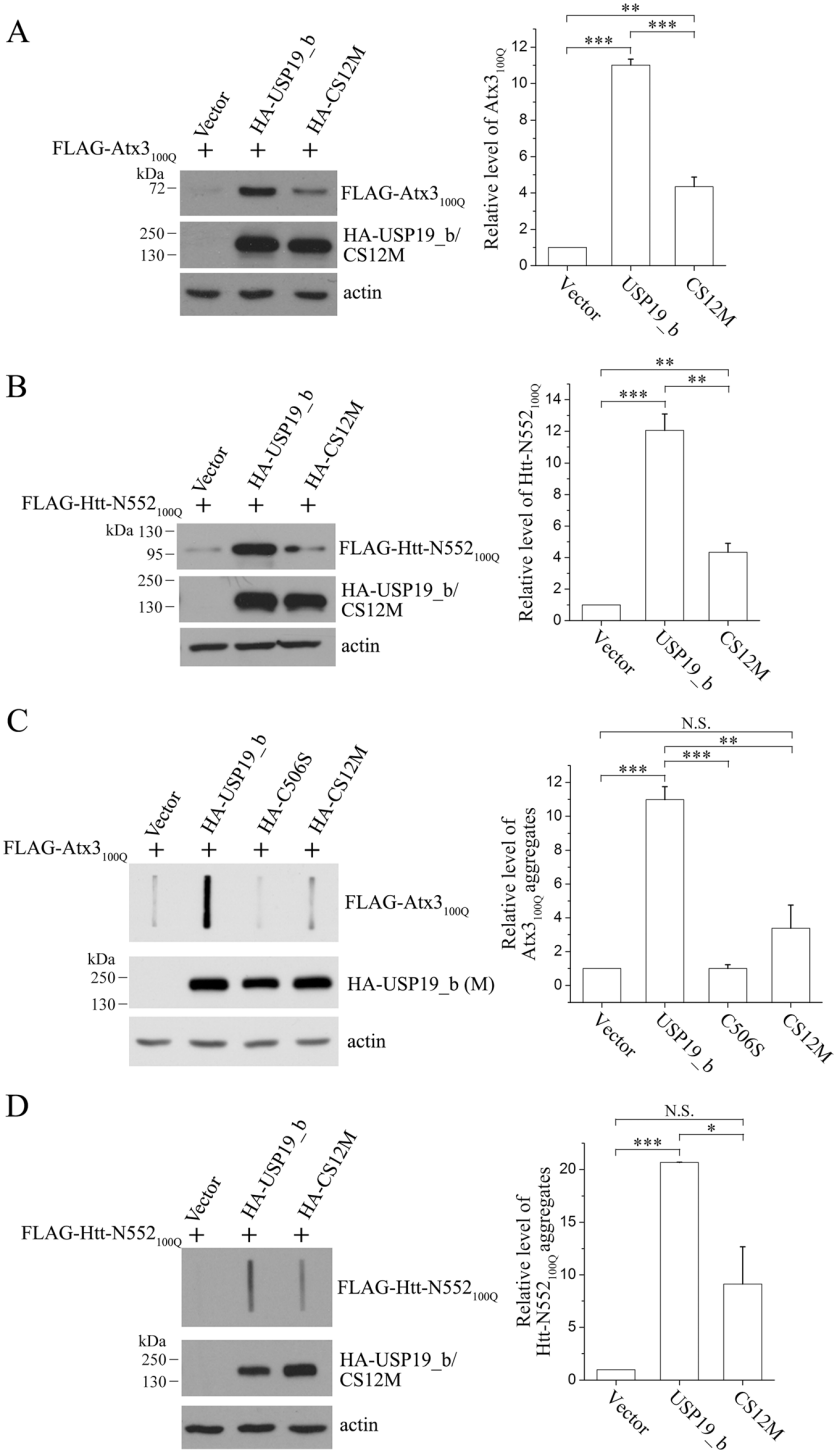
CS-domain mutation eliminates the promoting effects of USP19_b on Atx3_100Q_ and Htt-N552_100Q_. **A** and **B**, Effects of CS-domain mutation on the protein levels of Atx3_100Q_ and Htt-N552_100Q_. FLAG-tagged Atx3_100Q_ (**A**) or Htt-N552_100Q_ (**B**) was co-transfected with HA-USP19_b or its CS12M mutant into HEK 293T cells. About 48 hrs after transfection, the total protein levels were analyzed by Western blotting with an anti-FLAG antibody. **C** and **D**, Effects of CS-domain mutation on the aggregates of Atx3_100Q_ and Htt-N552_100Q_. The SDS-resistant aggregates of Atx3_100Q_ (**C**) and Htt-N552_100Q_ (**D**) were analyzed by filter trap. Data were quantitated with the relative band intensities and presented as Mean ± SEM (n = 3). *, p < 0.05; **, p < 0.01; ***, p < 0.001; N.S., no significance.

To verify this finding that USP19_b up-regulates the protein levels of polyQ proteins and thus promotes aggregation of their polyQ-expanded species through HSP90, we determined the effects of the USP19_b mutants on the total amounts and the aggregates of Atx3_100Q_ and Htt-N552_100Q_ in cell lysates. The data clearly indicated that the CS-domain mutation in USP19_b significantly abolished its effect to increase the total amounts (Fig 6A and 6B) and the aggregates (Fig 6C and 6D) of these two substrates. Again, USP19_b could up-regulate the protein levels of overexpressed Atx3 and Htt-N552 with normal polyQ lengths, whereas the CS-domain mutant significantly reduced this effect (S6 Fig). Collectively, the soluble and aggregated forms of the polyQ-expanded proteins are modulated by USP19_b through the chaperone system especially HSP90.

### USP19_b deubiquitinates the polyQ-expanded proteins through the HSP90 functionality

**Fig 7:**
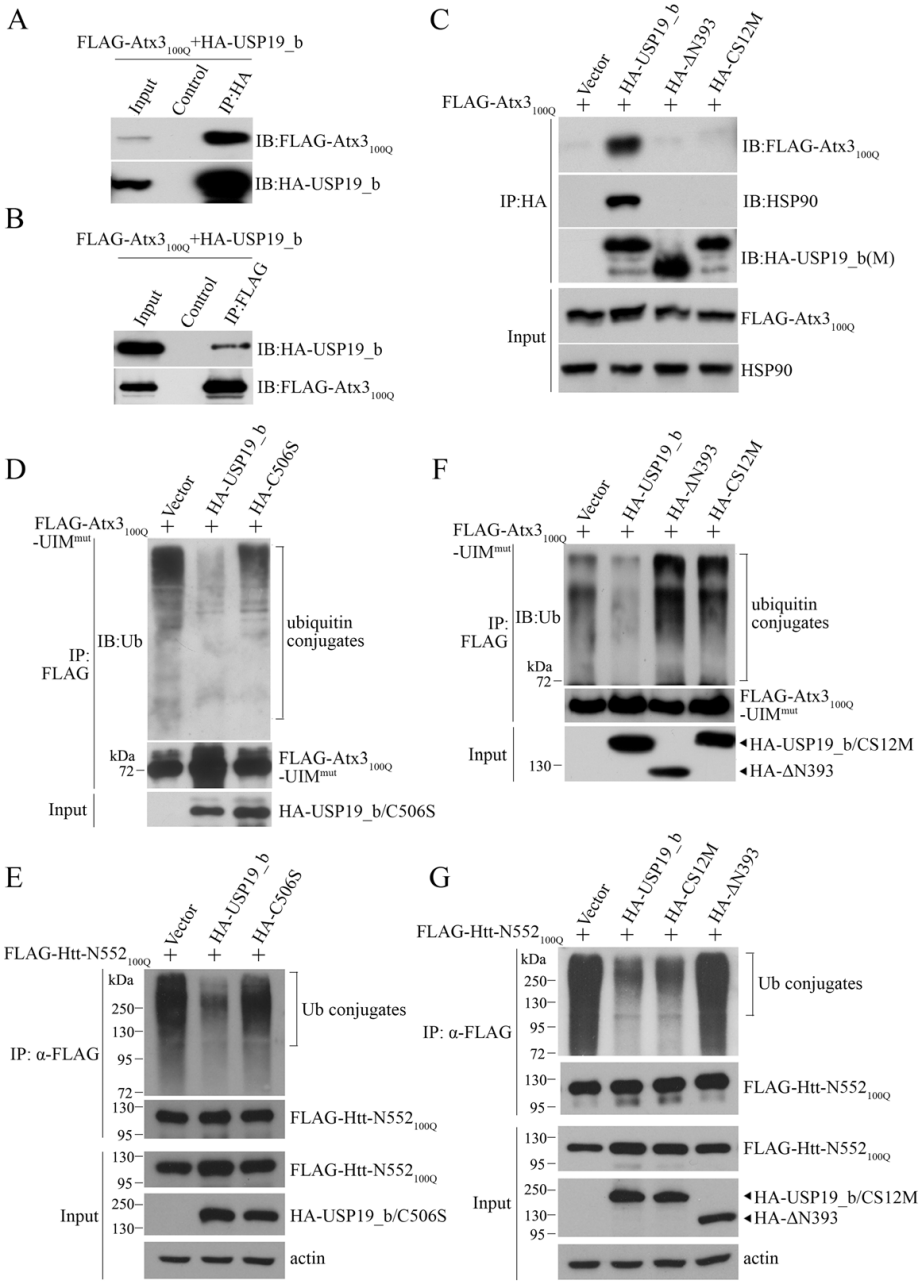
USP19_b regulates the ubiquitination status of Atx3_100Q_ and Htt-N552_100Q_ through the HSP90 chaperone. **A** and **B**, Co-IP experiment showing the association of USP19_b with Atx3_100Q_. HEK 293T cells were transfected with HA-USP19_b and FLAG-Atx3_100Q_, and the co-IP experiments were carried out by using anti-HA antibody (**A**) or anti-FLAG antibody (**B**). Control, IP by using protein A/G only. **C**, Co-IP experiment showing USP19_b associates with Atx3_100Q_ mediated by endogenous HSP90. The CS-domain mutants (ΔN393 and CS12M) were as controls. The cell lysates were prepared for Western blotting with anti-HA antibody for USP19_b and its mutants, and anti-FLAG antibody for Atx3_100Q_ and an antibody against endogenous HSP90. ΔN393, the CS-domain mutant of USP19_b with deletion of the N-terminal 393 residues. **D** and **E**, USP19_b reduces the ubiquitination levels of Atx3_100Q_-UIM^mut^ (**D**) and Htt-N552_100Q_ (**E**). HEK 293T cells were transfected with FLAG-tagged Atx3_100Q_-UIM^mut^ or Htt-N552_100Q_ and HA-USP19_b or its C506S mutant. The FLAG-tagged proteins were immunoprecipitated by using an anti-FLAG antibody, and subjected to Western blotting with the antibodies against ubiquitin and FLAG, respectively. **F** and **G**, Effects of CS-domain mutation (ΔN393 and CS12M) in USP19_b on the ubiquitination levels of Atx3_100Q_-UIM^mut^ (**F**) and Htt-N552_100Q_ (**G**).

To understand how HSP90 mediates the regulation of the polyQ-expanded substrates by USP19, we performed co-IP experiment on Atx3_100Q_ and USP19_b. Atx3_100Q_ could be immunoprecipitated by USP19_b (Fig 7A), and vice versa (Fig 7B). Moreover, wild-type USP19_b immunoprecipitated both Atx3_100Q_ and endogenous HSP90 (Fig 7C), whereas the CS domain-deleted (ΔN393) and mutated (CS12M) variants could not, suggesting that recruitment of Atx3_100Q_ by USP19_b is directly mediated by HSP90.

Considering that USP19 is a ubiquitin-specific protease, we next investigated the ubiquitination levels of Atx3_100Q_ and Htt-N552_100Q_ affected by USP19_b. Since isoform I of Atx3 contains two ubiquitin-interacting motifs (UIMs) that may probably pull down other ubiquitinated proteins, we prepared mutations in the UIM regions of Atx3 (Atx3_100Q_-UIM^mut^, S236A/S256A) [36, 37]. When co-transfected with wild-type USP19_b, the ubiquitination level of Atx3_100Q_ was dramatically decreased, while this effect was eliminated upon expression of the C506S mutant (Fig 7D), but no such effect was observed upon co-expression of USP5 (data not shown). This implies that USP19_b may function as a DUB for regulating the ubiquitination levels of the polyQ-expanded substrates. Similar effect was also observed in Htt-N552_100Q_ (Fig 7E). On the other hand, two CS-domain mutants of USP19_b, ΔN393 and CS12M, which could not associate with HSP90 (Fig 7C), had no such reducing effect on the ubiquitination levels (Fig 7F and 7G). Taken together, the HSP90 chaperone is probably involved in the deubiquitination of the polyQ-expanded substrates by US19_b.

## Discussion

USP19 is a DUB probably involved in regulating the ubiquitination status of substrate proteins. There are mainly two alternative variants of USP19 identified in human, which have distinct C-termini. The most widely studied isoform USP19_a contains a TMD for anchoring the protein on ER membrane [15], and enhances stabilities of several key proteins or enzymes [21–23]. We studied the regulatory function of cytoplasmic USP19 in defining the fate of the cytoplasm-resident polyQ-expanded proteins, Atx3 and Htt-N552, in cell models. We have revealed that USP19_b up-regulates the protein levels of Atx3_100Q_ and Htt-N552_100Q_ and consequently promotes their aggregation. Although we could not exclude the possibility that other aggregation-prone or disease-related proteins are regulated by USP19_b, our data provide supporting evidence that USP19_b modulates aggregation of the polyQ-expanded proteins through HSP90. Note that the ER-resident isoform USP19_a also has this regulatory function. We speculate that only the C-terminal tail of USP19_a is located in the ER lumen, whereas most of the functional part is towards the cytoplasm that renders it to act on the cytoplasmic substrates [15, 20]. Both forms of USP19 specifically interact with HSP90 through their N-terminal CS domains and then associate with CHIP via HSP90. Thus, considering the regulatory role of HSP90 on misfolded proteins [38], it is suggestive that USP19 is involved in quality control for the cytoplasmic misfolded proteins through the HSP90 chaperone.

The major finding of this work is that cytoplasmic USP19 can promote aggregation of the polyQ-expanded Atx3 and Htt proteins by up-regulating their protein levels. Although USP19_b also up-regulates the protein levels of endogenous Atx3, and overexpressed Atx3_22Q_ and Htt-N552_18Q_, we have not observed the aggregates or inclusions formed by overexpressed proteins with normal polyQ lengths (Atx3_22Q_, Htt-N552_18Q_) in the cultured cells, even in the presence of overexpressed USP19_b (Fig 2). This means that expansion of polyQ tract provides a potential for Atx3 and Htt-N552 to form insoluble aggregates; and cooperating with HSP90, USP19 aggravates the aggregation of these polyQ-expanded proteins and enhances their cytotoxicities.

We have demonstrated that USP19_b specifically interacts with HSP90, where HSP90 recruits CHIP and the misfolded substrate to delicately regulate ubiquitination status of the substrate. The HSP90 molecule is a hub for protein triage and maturation of a variety of client proteins [38], which can rescue cells from toxicities of the polyQ-expanded proteins and prevent degeneration [39]. Inhibition of HSP90 can result in the degradation of its substrates including polyQ-expanded proteins [35, 40]. We have demonstrated that the HSP90 inhibitor 17-AAG can decrease the protein level of Atx3_100Q_ or Htt-N552_100Q_ and consequently alleviate their aggregation. The degradation of HSP90 substrates induced by 17-AAG treatment is probably due to inhibition of HSP90 activity and disruption of HSP90-substrate interaction [40]. Furthermore, 17-AAG can activate heat shock response through dissociation of heat-shock transcription factor (HSF-1) from HSP90 and induction of HSP70 or other HSPs mediated by HSF-1 [41]. Thus HSP70 is also probably involved in the degradation of HSP90 substrates after 17-AAG treatment. This implicates a tight connection and a delicate balance between the molecular chaperone and ubiquitin-proteasome systems [6].

USP19 is a multi-domain protein functioning both as a DUB and a co-chaperone protein; its cytoplasmic isoform can regulate misfolded polyQ-expanded proteins through the HSP90 chaperone. HSP90 recruits the misfolded substrate for refolding or rescue, which may promote stabilization of the substrate and consequently up-regulate its biological function. With the help of HSP90, CHIP can ubiquitinate the substrate that fails to be refolded for degradation in proteasome, which results in reduction of the protein level of the substrate. On the other hand, the USP domain of USP19 deubiquitinates the substrate and prevents it from degradation, which leads to aggregation of the misfolded substrate. Thus, the dual function of USP19 may play an important role in orchestrating the client proteins. This mechanism of triage decision through the HSP90 chaperone may have significance in balancing the refolding, degradation and aggregation of the polyQ-expanded proteins. Breakdown of the homeostasis by physiological stress or genetic mutations is potentially the pathological cause of the polyQ diseases [32], while targeting this homeostatic pathway by small-molecule compounds may provide a promising therapeutic strategy for these aggravating diseases.

## Supporting Information

S1 Fig The USP19_b isoform is a cytoplasmic ubiquitin-specific protease. (PDF)

S2 Fig Domain architectures of isoform I of ataxin-3 (Atx3) and N-terminal huntingtin (Htt-N552, residues 1–552). (PDF)

S3 Fig USP19_b increases the protein levels of Atx3 and Htt-N552. (PDF)

S4 Fig Contrasting experiments for the regulatory effects of USP19_b on the substrate proteins. (PDF)

S5 Fig Supernatant/pellet fractionation showing that USP19_b up-regulates aggregation of Atx3_100Q_ in transiently overexpressing cells. (PDF)

S6 Fig CS-domain mutation in USP19_b eliminates the promoting effects on the protein levels of overexpressed Atx3_22Q_ and Htt-N552_18Q_. (PDF)
