# Supplementary material for: Cytoplasmic Ubiquitin-Specific Protease 19 (USP19) Modulates Aggregation of Polyglutamine-Expanded Ataxin-3 and Huntingtin through the HSP90 Chaperone
Source: PLoS One. 2016 Jan 25;11(1):e0147515. doi: 10.1371/journal.pone.0147515 (PMC4726498; doi:10.1371/journal.pone.0147515)
Supplement: S1 Fig — (PDF) [file pone.0147515.s001.pdf]

S1 Fig

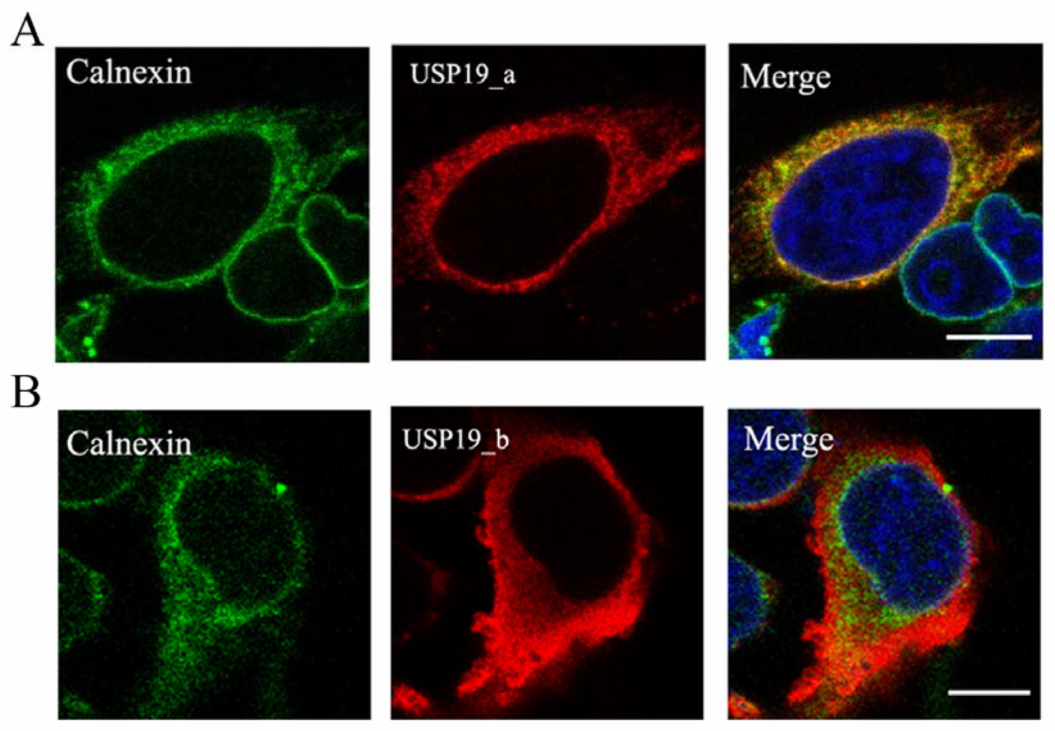

S1 Fig. The USP19_b isoform is a cytoplasmic ubiquitin-specific protease. A, Immunofluorescence microscopy imaging showing cellular localization of USP19_a. **B**, Cytoplasmic localization of USP19_b. HA-tagged USP19_a or USP19_b was transiently transfected into HEK 293T cells, and 36 hrs later, the cells were fixed for imaging. USP19_a and USP19_b were visualized with anti-HA antibody (red), the ER location was indicated with an antibody against calnexin (green), and the nucleus was stained by Hoechst (blue). Scalar bar = 10 µm.
