# Supplementary material for: Cytoplasmic Ubiquitin-Specific Protease 19 (USP19) Modulates Aggregation of Polyglutamine-Expanded Ataxin-3 and Huntingtin through the HSP90 Chaperone
Source: PLoS One. 2016 Jan 25;11(1):e0147515. doi: 10.1371/journal.pone.0147515 (PMC4726498; doi:10.1371/journal.pone.0147515)
Supplement: S2 Fig — (PDF) [file pone.0147515.s002.pdf]

S2 Fig

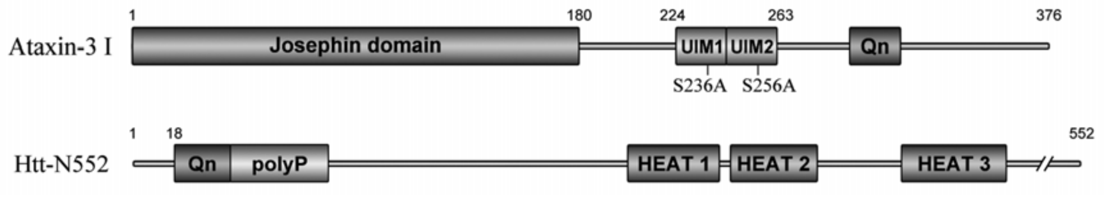

S2 Fig. Domain architectures of isoform I of ataxin-3 (Atx3) and N-terminal huntingtin (Htt-N552, residues 1 - 552). Qn, polyQ tract; UIM, ubiquitin-interacting motif; S236A and S256A, the mutation sites in the UIM region.
