# Supplementary material for: Cytoplasmic Ubiquitin-Specific Protease 19 (USP19) Modulates Aggregation of Polyglutamine-Expanded Ataxin-3 and Huntingtin through the HSP90 Chaperone
Source: PLoS One. 2016 Jan 25;11(1):e0147515. doi: 10.1371/journal.pone.0147515 (PMC4726498; doi:10.1371/journal.pone.0147515)
Supplement: S3 Fig — (PDF) [file pone.0147515.s003.pdf]

S3 Fig

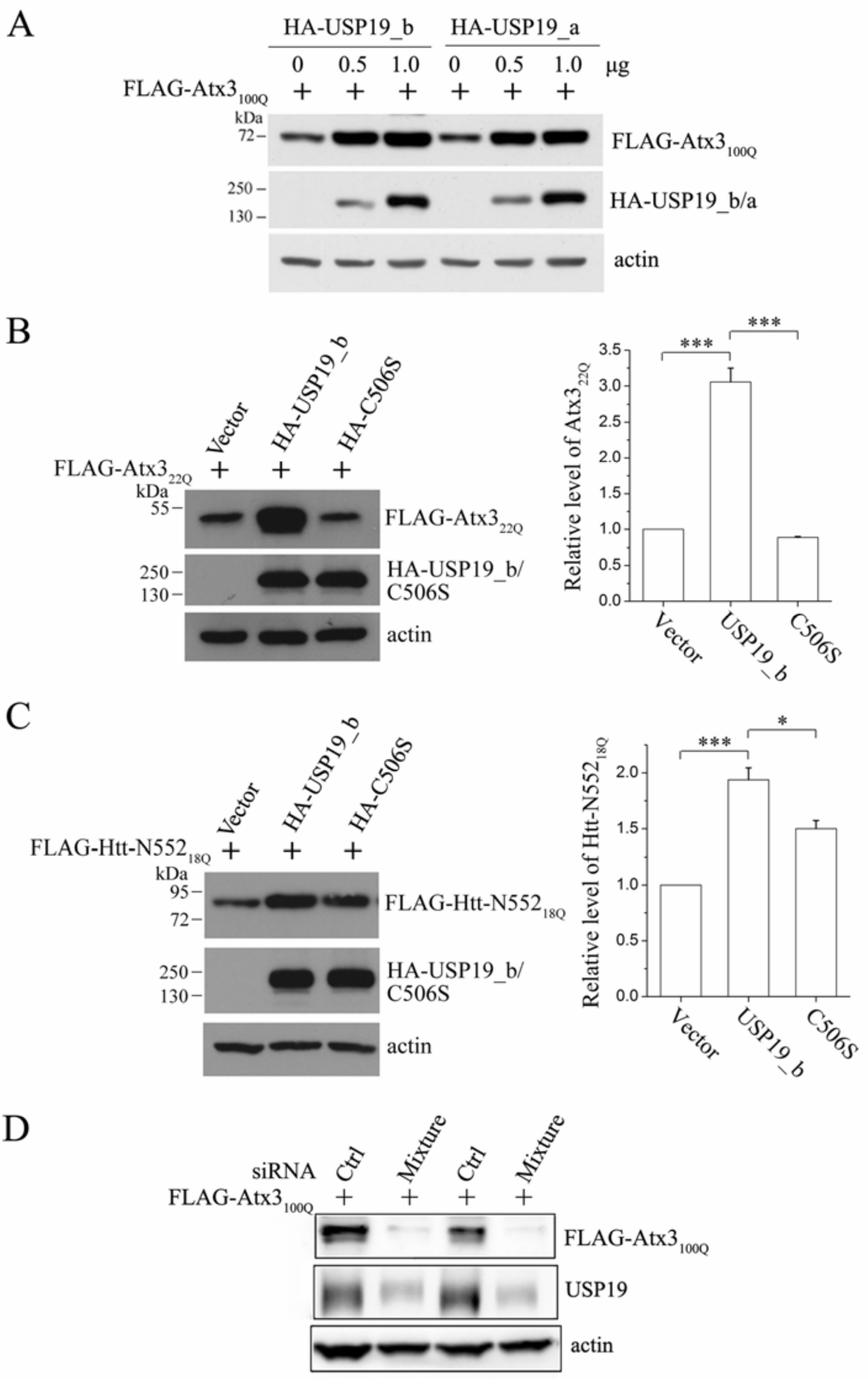

S3 Fig. USP19_b increases the protein levels of Atx3 and Htt-N552. A, Dose-dependent experiment on the protein level of $Atx3_{100Q}$ enhanced by USP19_b or USP19_a. FLAG-$Atx3_{100Q}$ was co-transfected with different doses of USP19_b or

USP19_a, and then the protein level of $Atx3_{100Q}$ was detected by Western blotting. **B** and **C**, Effects of USP19_b on the protein levels of overexpressed $Atx3_{22Q}$ (**B**) and Htt-$N552_{18Q}$ (**C**). Data were presented as Mean ± SEM (n = 3). *, $p < 0.05$; ***, $p < 0.001$. **D,** Knockdown of USP19 reduces the protein level of $Atx3_{100Q}$ in human retinal pigmentepithelial (RPE1) cells. Cells were transfected with FLAG-$Atx3_{100Q}$ and USP19 siRNA. After 72 hrs, the cells were harvested and the lysates were subjected to Western blotting with the indicated antibodies. Ctrl, VSVG siRNA; mixture, 1#, 2# and 3# siRNAs against USP19.
