# Supplementary material for: Cytoplasmic Ubiquitin-Specific Protease 19 (USP19) Modulates Aggregation of Polyglutamine-Expanded Ataxin-3 and Huntingtin through the HSP90 Chaperone
Source: PLoS One. 2016 Jan 25;11(1):e0147515. doi: 10.1371/journal.pone.0147515 (PMC4726498; doi:10.1371/journal.pone.0147515)
Supplement: S4 Fig — (PDF) [file pone.0147515.s004.pdf]

**S4 Fig**

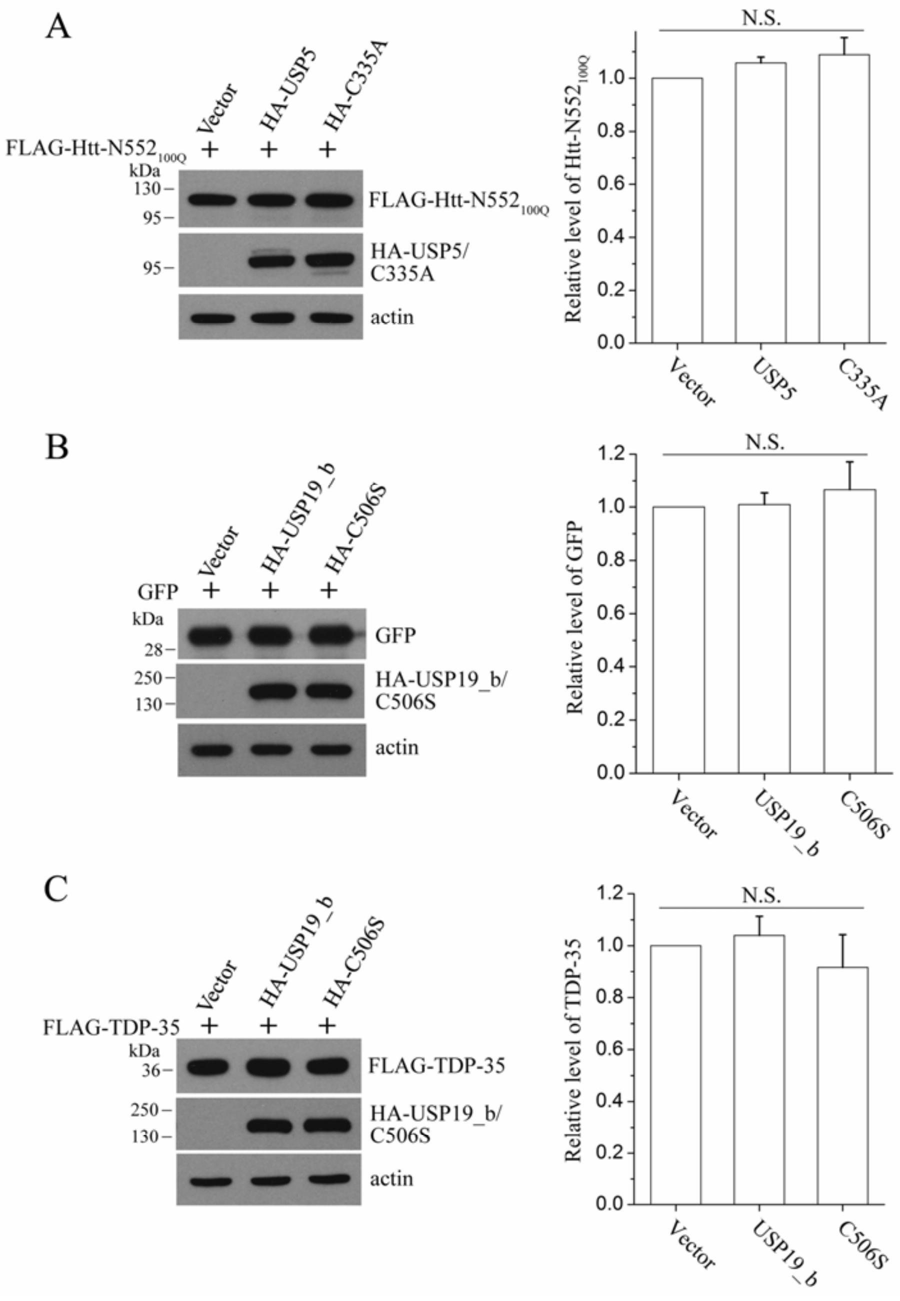


**S4 Fig. Contrasting experiments for the regulatory effects of USP19_b on substrate proteins. A,** Effect of USP5 on the protein level of Htt-N552$_{100Q}$. FLAG-tagged Htt-N552$_{100Q}$ was co-transfected with HA-USP5 or its active-site

mutant (C335A) into HEK 293T cells. **B** and **C,** Effects of USP19_b on the protein levels of GFP and TDP-35. GFP (**B**) or TDP-35 (**C**) was co-transfected with HA-USP19_b or its C506S mutant into HEK 293T cells. About 48 hrs after transfection, the total protein levels were analyzed by Western blotting with indicated antibodies. Data were presented as Mean ± SEM (n = 3). N.S., no significance.
