# Supplementary material for: Cytoplasmic Ubiquitin-Specific Protease 19 (USP19) Modulates Aggregation of Polyglutamine-Expanded Ataxin-3 and Huntingtin through the HSP90 Chaperone
Source: PLoS One. 2016 Jan 25;11(1):e0147515. doi: 10.1371/journal.pone.0147515 (PMC4726498; doi:10.1371/journal.pone.0147515)
Supplement: S5 Fig — (PDF) [file pone.0147515.s005.pdf]

S5 Fig

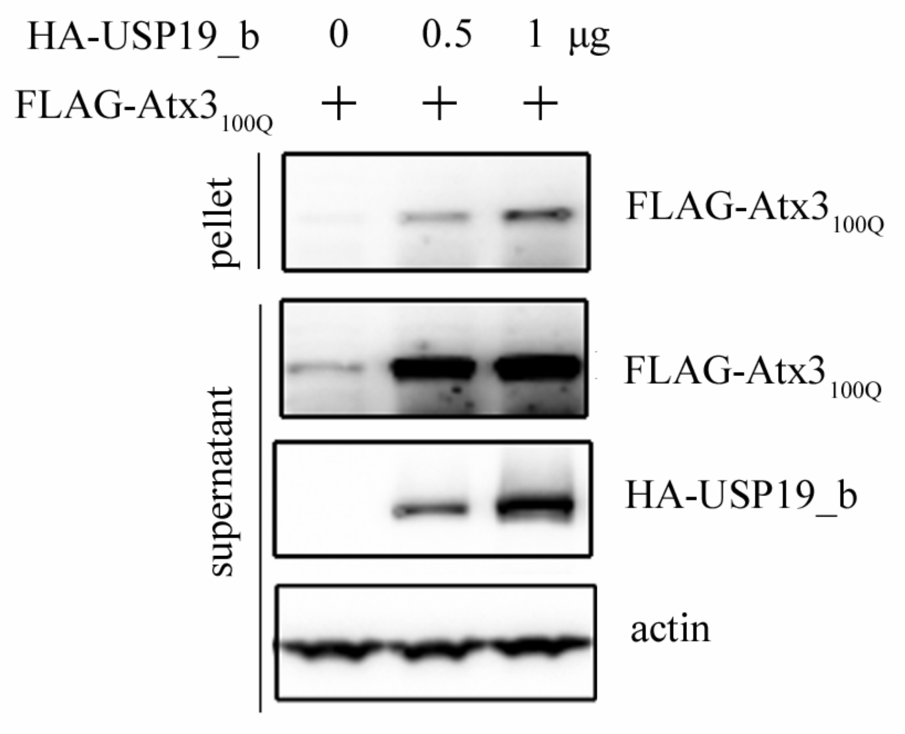

S5 Fig. Supernatant/pellet fractionation showing that USP19_b up-regulates aggregation of Atx3$_{100Q}$ in transiently overexpressing cells. HEK 293T cells were transfected with equal amount of FLAG-Atx3$_{100Q}$ and increasing amount of HA-USP19_b, and then the cell lysates were fractionalized into supernatant (S) and pellet (P).
