# Supplementary material for: Cytoplasmic Ubiquitin-Specific Protease 19 (USP19) Modulates Aggregation of Polyglutamine-Expanded Ataxin-3 and Huntingtin through the HSP90 Chaperone
Source: PLoS One. 2016 Jan 25;11(1):e0147515. doi: 10.1371/journal.pone.0147515 (PMC4726498; doi:10.1371/journal.pone.0147515)
Supplement: S6 Fig — (PDF) [file pone.0147515.s006.pdf]

S6 Fig

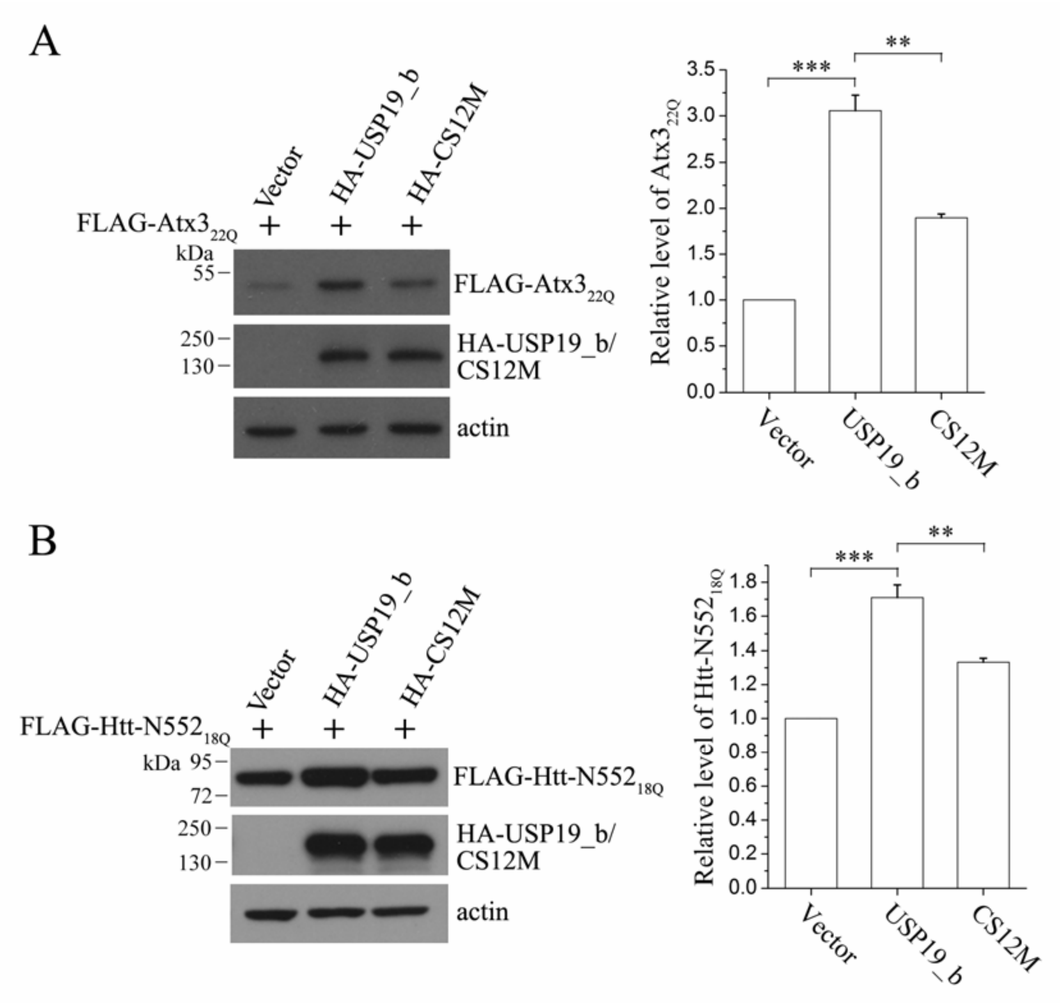

S6 Fig. CS-domain mutation in USP19_b eliminates the promoting effects on the protein levels of overexpressed Atx3$_{22Q}$ and Htt-N552$_{18Q}$. FLAG-tagged Atx3$_{22Q}$ (**A**) or Htt-N552$_{18Q}$ (**B**) was co-transfected with HA-USP19_b or its CS12M mutant into HEK 293T cells. About 48 hrs after transfection, the total protein levels were analyzed by Western blotting with an anti-FLAG antibody.
